# Supplementary material for: Unveiling the Bioactive Potential of the Invasive Jellyfish Phyllorhiza punctata Through Integrative Transcriptomic and Proteomic Analyses
Source: Biomolecules. 2025 Aug 4;15(8):1121. doi: 10.3390/biom15081121 (PMC12383608; doi:10.3390/biom15081121)
Supplement: Supplementary file 1 [file biomolecules-15-01121-s001.zip › Suplementary Material/FileS3/PEPFOLD4/XP_065070945_16501/clusters.html]

 PEP-FOLD clusters


Download archive of all models  here   

|  |  |  |
| --- | --- | --- |
| # | file | sOPEP |
| # cluster 1 - Size: 1 |  |  |
|  | XP\_065070945\_16501-model1 | -10.5949 |
| # cluster 2 - Size: 1 |  |  |
|  | XP\_065070945\_16501-model2 | -9.94155 |
| # cluster 3 - Size: 3 |  |  |
|  | XP\_065070945\_16501-model3 | -9.00804 |
|  | XP\_065070945\_16501-model3.1 | -6.2957 |
|  | XP\_065070945\_16501-model3.2 | -6.25839 |
| # cluster 4 - Size: 2 |  |  |
|  | XP\_065070945\_16501-model4 | -8.42943 |
|  | XP\_065070945\_16501-model4.1 | -7.06457 |
| # cluster 5 - Size: 2 |  |  |
|  | XP\_065070945\_16501-model5 | -8.2735 |
|  | XP\_065070945\_16501-model5.1 | -6.27528 |
| # cluster 6 - Size: 8 |  |  |
|  | XP\_065070945\_16501-model6 | -7.95971 |
|  | XP\_065070945\_16501-model6.1 | -7.34516 |
|  | XP\_065070945\_16501-model6.2 | -7.21412 |
|  | XP\_065070945\_16501-model6.3 | -7.13629 |
|  | XP\_065070945\_16501-model6.4 | -6.28314 |
|  | XP\_065070945\_16501-model6.5 | -6.01253 |
|  | XP\_065070945\_16501-model6.6 | -5.88137 |
|  | XP\_065070945\_16501-model6.7 | -4.98772 |
| # cluster 7 - Size: 1 |  |  |
|  | XP\_065070945\_16501-model7 | -7.62839 |
| # cluster 8 - Size: 3 |  |  |
|  | XP\_065070945\_16501-model8 | -7.49253 |
|  | XP\_065070945\_16501-model8.1 | -6.20467 |
|  | XP\_065070945\_16501-model8.2 | -5.98667 |
| # cluster 9 - Size: 1 |  |  |
|  | XP\_065070945\_16501-model9 | -7.49198 |
| # cluster 10 - Size: 8 |  |  |
|  | XP\_065070945\_16501-model10 | -7.39619 |
|  | XP\_065070945\_16501-model10.1 | -7.13813 |
|  | XP\_065070945\_16501-model10.2 | -7.12804 |
|  | XP\_065070945\_16501-model10.3 | -6.4314 |
|  | XP\_065070945\_16501-model10.4 | -6.21356 |
|  | XP\_065070945\_16501-model10.5 | -6.11853 |
|  | XP\_065070945\_16501-model10.6 | -5.65876 |
|  | XP\_065070945\_16501-model10.7 | -5.64182 |
| # cluster 11 - Size: 2 |  |  |
|  | XP\_065070945\_16501-model11 | -7.3375 |
|  | XP\_065070945\_16501-model11.1 | -6.24978 |
| # cluster 12 - Size: 1 |  |  |
|  | XP\_065070945\_16501-model12 | -7.33458 |
| # cluster 13 - Size: 1 |  |  |
|  | XP\_065070945\_16501-model13 | -7.18664 |
| # cluster 14 - Size: 3 |  |  |
|  | XP\_065070945\_16501-model14 | -7.18095 |
|  | XP\_065070945\_16501-model14.1 | -6.07483 |
|  | XP\_065070945\_16501-model14.2 | -6.00982 |
| # cluster 15 - Size: 4 |  |  |
|  | XP\_065070945\_16501-model15 | -6.95814 |
|  | XP\_065070945\_16501-model15.1 | -6.84099 |
|  | XP\_065070945\_16501-model15.2 | -6.59448 |
|  | XP\_065070945\_16501-model15.3 | -4.93842 |
| # cluster 16 - Size: 8 |  |  |
|  | XP\_065070945\_16501-model16 | -6.89286 |
|  | XP\_065070945\_16501-model16.1 | -6.67337 |
|  | XP\_065070945\_16501-model16.2 | -6.66356 |
|  | XP\_065070945\_16501-model16.3 | -6.50495 |
|  | XP\_065070945\_16501-model16.4 | -5.87442 |
|  | XP\_065070945\_16501-model16.5 | -5.80422 |
|  | XP\_065070945\_16501-model16.6 | -5.7901 |
|  | XP\_065070945\_16501-model16.7 | -5.71317 |
| # cluster 17 - Size: 1 |  |  |
|  | XP\_065070945\_16501-model17 | -6.87159 |
| # cluster 18 - Size: 1 |  |  |
|  | XP\_065070945\_16501-model18 | -6.80015 |
| # cluster 19 - Size: 3 |  |  |
|  | XP\_065070945\_16501-model19 | -6.79065 |
|  | XP\_065070945\_16501-model19.1 | -6.14915 |
|  | XP\_065070945\_16501-model19.2 | -5.92527 |
| # cluster 20 - Size: 1 |  |  |
|  | XP\_065070945\_16501-model20 | -6.73076 |
| # cluster 21 - Size: 2 |  |  |
|  | XP\_065070945\_16501-model21 | -6.692 |
|  | XP\_065070945\_16501-model21.1 | -6.24213 |
| # cluster 22 - Size: 1 |  |  |
|  | XP\_065070945\_16501-model22 | -6.64144 |
| # cluster 23 - Size: 2 |  |  |
|  | XP\_065070945\_16501-model23 | -6.61854 |
|  | XP\_065070945\_16501-model23.1 | -6.46516 |
| # cluster 24 - Size: 2 |  |  |
|  | XP\_065070945\_16501-model24 | -6.50884 |
|  | XP\_065070945\_16501-model24.1 | -6.20299 |
| # cluster 25 - Size: 3 |  |  |
|  | XP\_065070945\_16501-model25 | -6.46301 |
|  | XP\_065070945\_16501-model25.1 | -5.91916 |
|  | XP\_065070945\_16501-model25.2 | -5.31929 |
| # cluster 26 - Size: 1 |  |  |
|  | XP\_065070945\_16501-model26 | -6.43971 |
| # cluster 27 - Size: 2 |  |  |
|  | XP\_065070945\_16501-model27 | -6.43707 |
|  | XP\_065070945\_16501-model27.1 | -6.05968 |
| # cluster 28 - Size: 1 |  |  |
|  | XP\_065070945\_16501-model28 | -6.41105 |
| # cluster 29 - Size: 1 |  |  |
|  | XP\_065070945\_16501-model29 | -6.35138 |
| # cluster 30 - Size: 1 |  |  |
|  | XP\_065070945\_16501-model30 | -6.34608 |
| # cluster 31 - Size: 3 |  |  |
|  | XP\_065070945\_16501-model31 | -6.29075 |
|  | XP\_065070945\_16501-model31.1 | -6.09912 |
|  | XP\_065070945\_16501-model31.2 | -5.7515 |
| # cluster 32 - Size: 2 |  |  |
|  | XP\_065070945\_16501-model32 | -6.13783 |
|  | XP\_065070945\_16501-model32.1 | -5.02218 |
| # cluster 33 - Size: 1 |  |  |
|  | XP\_065070945\_16501-model33 | -6.02048 |
| # cluster 34 - Size: 2 |  |  |
|  | XP\_065070945\_16501-model34 | -5.84956 |
|  | XP\_065070945\_16501-model34.1 | -5.05392 |
| # cluster 35 - Size: 1 |  |  |
|  | XP\_065070945\_16501-model35 | -5.79837 |
| # cluster 36 - Size: 1 |  |  |
|  | XP\_065070945\_16501-model36 | -5.75755 |
| # cluster 37 - Size: 1 |  |  |
|  | XP\_065070945\_16501-model37 | -5.71201 |
| # cluster 38 - Size: 1 |  |  |
|  | XP\_065070945\_16501-model38 | -5.64779 |
| # cluster 39 - Size: 1 |  |  |
|  | XP\_065070945\_16501-model39 | -5.64701 |
| # cluster 40 - Size: 1 |  |  |
|  | XP\_065070945\_16501-model40 | -5.64365 |
| # cluster 41 - Size: 1 |  |  |
|  | XP\_065070945\_16501-model41 | -5.63421 |
| # cluster 42 - Size: 1 |  |  |
|  | XP\_065070945\_16501-model42 | -5.62482 |
| # cluster 43 - Size: 1 |  |  |
|  | XP\_065070945\_16501-model43 | -5.56089 |
| # cluster 44 - Size: 2 |  |  |
|  | XP\_065070945\_16501-model44 | -5.51624 |
|  | XP\_065070945\_16501-model44.1 | -5.23187 |
| # cluster 45 - Size: 2 |  |  |
|  | XP\_065070945\_16501-model45 | -5.46713 |
|  | XP\_065070945\_16501-model45.1 | -5.18122 |
| # cluster 46 - Size: 1 |  |  |
|  | XP\_065070945\_16501-model46 | -5.4533 |
| # cluster 47 - Size: 1 |  |  |
|  | XP\_065070945\_16501-model47 | -5.4282 |
| # cluster 48 - Size: 1 |  |  |
|  | XP\_065070945\_16501-model48 | -5.38454 |
| # cluster 49 - Size: 1 |  |  |
|  | XP\_065070945\_16501-model49 | -5.23769 |
| # cluster 50 - Size: 1 |  |  |
|  | XP\_065070945\_16501-model50 | -5.00983 |
| # cluster 51 - Size: 1 |  |  |
|  | XP\_065070945\_16501-model51 | -4.88444 |
| # cluster 52 - Size: 1 |  |  |
|  | XP\_065070945\_16501-model52 | -4.87579 |
| # cluster 53 - Size: 1 |  |  |
|  | XP\_065070945\_16501-model53 | -4.71763 |
|  |  |  |
